# Spiral Microchannels with Trapezoidal Cross Section Fabricated by Femtosecond Laser Ablation in Glass for the Inertial Separation of Microparticles

**DOI:** 10.3390/mi9040171

**Published:** 2018-04-09

**Authors:** Ala’aldeen Al-Halhouli, Wisam Al-Faqheri, Baider Alhamarneh, Lars Hecht, Andreas Dietzel

**Affiliations:** 1 NanoLab, School of Applied Technical Sciences, German Jordanian University (GJU), Amman 11180, Jordan; Wisam.AlFakhri@gju.edu.jo (W.A.-F.); bayderh@gmail.com (B.A.); 2 Institut für Mikrotechnik, Technische Universität Braunschweig, Braunschweig 38124, Germany; lars.hecht@tu-braunschweig.de (L.H.); a.dietzel@tu-braunschweig.de (A.D.)

**Keywords:** microfluidics, 3D fabrication, spiral channel, particles separation, femtosecond laser

## Abstract

The fabrication and testing of spiral microchannels with a trapezoidal cross section for the passive separation of microparticles is reported in this article. In contrast to previously reported fabrication methods, the fabrication of trapezoidal spiral channels in glass substrates using a femtosecond laser is reported for the first time in this paper. Femtosecond laser ablation has been proposed as an accurate and fast prototyping method with the ability to create 3D features such as slanted-base channels. Moreover, the fabrication in borosilicate glass substrates can provide high optical transparency, thermal resistance, dimensional stability, and chemical inertness. Post-processing steps of the laser engraved glass substrate are also detailed in this paper including hydrogen fluoride (HF) dipping, chemical cleaning, surface activation, and thermal bonding. Optical 3D images of the fabricated chips confirmed a good fabrication accuracy and acceptable surface roughness. To evaluate the particle separation function of the microfluidic chip, 5 μm, 10 μm, and 15 μm particles were focused and recovered from the two outlets of the spiral channel. In conclusion, the new chemically inert separation chip can be utilized in biological or chemical processes where different sizes of cells or particles must be separated, i.e., red blood cells, circulating tumor cells, and technical particle suspensions.

## 1. Introduction

In various biomedical, biotechnological, and pharmaceutical applications, the separation and recovery of specific particles from the background mixture is an indispensable step that usually comes directly before multi post-processing stages. The throughput and the produced purity of this step is a key factor in the results of the following processes. Therefore, several microfluidic-based mechanisms have already been developed and tested for the separation of particles and/or cells based on their unique characteristics (e.g., geometry, physical, chemical, or genetic properties). Such mechanisms are mostly classified according to operation principle, under active or passive methods [1]. Active methods including dielectrophoresis (DEP) and separation with magnetic fields are methods that utilize external force to separate between the different particles. In contrast, passive methods are defined as the separation of particles without installing an external force or trigger such as particle sedimentation, immunoaffinity, and separation based on physical properties (filtering, and inertial separation). Recently, a number of review articles have highlighted the available mechanisms of particle/cell isolation using different types of microfluidic platforms [2,3,4,5,6,7].

The separation of particles passively has shown numerous advantages including the low setup complexity and cost, high throughput, portability, and purity [1]. Inertial microfluidics are among the highly developed passive methods that employ the physical properties of particles (mainly particle size) in the separation and recovery process. In this method, a micro-scaled curved or multi-turn spiral channel is implemented to produce an inertial effect on particles/cells [8,9,10,11,12,13,14,15,16,17,18]. When a sample with randomly dispersed particles of different sizes is injected into such a spiral microfluidic channel, lift and drag forces will drive particles in different directions and in magnitudes proportional to the particle size [8]. With respect to the implemented channel dimensions (width and height), larger particles have a higher lift effect forcing them to migrate towards the inner wall (wall closer to the spiral center). In contrast, the dominant drag force effect on smaller particles forces them to migrate and focus near the outer wall of the channel (wall closer to the spiral outer edge) [1,8]. Therefore, by implementing proper channel dimensions and liquid flow rate, different size particles can be passively separated and recovered from different outlets of the spiral channel.

Spiral microfluidic channels with different dimensions, number of turns, and cross-sectional shapes were implemented for the separation and recovery of different types of particles and cells. Papautsky et al. [9,10,11] and the Lim and Han groups [12,13,14,15,16,17] have all published different papers on particle/cell separation using spiral-shaped microchannels. Various spiral microfluidic designs were proposed including spiral-turns with numbers ranging from 2 to 10, channel widths between 100 μm and 500 μm, and channel depths between 50 μm and 170 μm. Moreover, sheath and sheath-less flow mechanisms were implemented to improve the output of the spiral channel. High throughputs of up to 1 million cells/min were achieved with a high recovery rate that could reach 90% in some designs. In other works, trapezoidal cross-sectional spiral channels were proposed to generate stronger Dean drag force [13,18]. The implementation of stacked spiral channels was also demonstrated to produce ultra-fast particle/cell separation platforms [17].

### PDMS vs. Glass for Spiral Fabrication

For the last two decades, soft lithography with polydimethylsiloxane (PDMS) has been used by most researchers in the field of microfluidics due to its low fabrication cost, simple setup, and fast prototyping capability [19,20,21,22,23]. Furthermore, PDMS is nontoxic, biocompatible, optically transparent, nonflammable, and has good chemical and thermal stability [19,23,24]. More importantly, PDMS can be bonded to itself and various materials (e.g., glass) after oxygen or plasma treatment. This is a very important feature for the fabrication of microfluidic platforms that mostly consist of a multi-layer configuration. PDMS molding is usually performed with a mold or master that contains the required microfluidic features fabricated using micro-machining or photolithography. Afterwards, a mixture of siloxane oligomer and curing agent (usually 10:1 mixing ratio) is poured on the mold, degassed to remove bubbles, and cured at 70 °C for one hour. Finally, the cured PDMS layer with the microfluidic features can be easily peeled off the mold then bonded to a cover layer [19]. Due to this simplicity in fabrication, PDMS has been used in most of the developed spiral microfluidic platforms for particle/cell separation and recovery. 

Despite the above highlighted advantages of PDMS microfabrication, there are some serious drawbacks. These include: (1) the mechanical softness of PDMS limits the achievable aspect ratio of micro-structures or the implementation of multi-layered designs; (2) PDMS can swell through a variety of organic solvents, thereby limiting the range of application in the chemical field; (3) PDMS is hydrophobic and can absorb drugs, proteins, and small hydrophobic molecules; (4) exhibits aging effects that reduce shelve life; and (5) finally, and most importantly in the field of particle separation using inertial microfluidics, due to a low Young’s modulus *E* (in the range of 0.5 to 4 MPa depending on curing conditions), PDMS can withstand a specific range of pressures (flow rates) [24,25,26]. Higher pressures can cause 5% to 10% elastic deformation in channel thickness depend on the implemented pressure and channel aspect ratio. This PDMS deformation can result in big differences between the expected and the actual flow rate [25]. According to a detailed study by Gervais et al., the expected flow rate in a rigid channel under 1 bar pressure is 270 μL·min^−1^, while the actual flow rate in a deforming channel with exactly the same dimensions is measured to be 1600 μL·min^−1^ [25]. This noticeable difference in flow rate can be the main source of error in the predicted focusing path of specific particle size in the inertial focusing platform. These are factors that have made PDMS successful in many academic laboratories, but not in industrial applications.

On the other hand, glass is considered as the gold standard in many microfluidic applications due to its high optical transparency, thermal resistance, dimensional stability for any aspect ratio (*E* = 62 GPa), suitability for multi-layer fabrication, and chemical inertness [19,24]. In addition, glass can be bonded to itself using thermal bonding and to PDMS by oxygen plasma treatment. Finally, microstructures can be easily realized in glass using chemical etching or ultra-short pulse laser ablation. On the downside, glass is not the cheapest fabrication materials as it can cost a little above 3 euros per one microfluidic chip that consists of two glass layers. In addition, access to a femtosecond laser machine and clean room facility can increase the cost-per-chip to 5 euros. Therefore, glass ablation is a viable approach in the rapid prototyping phase where a limited number of microfluidic chips is required (<500). However, this method is not the best choice for a mass production phase (100,000 or above). In terms of fabrication time, etching of a single spiral channel chip using femtosecond laser can take around 4 min, and the total preparation/cleaning (around 30 min) and thermal bonding (6 h) is 6 h and 35 min. It is worth mentioning here that 3D stereo-lithographic (SL) is an additive manufacturing method that widely used to produce 3D microfluidic structures. This method can produce good fabrication resolution and acceptable fabrication time, but is too limited by the device speed and space [27,28]. Moreover, good dimensional stability, chemical inertness, and acceptable optical transparency can be produced using this method. Hot embossing is another fabrication method that can be implemented to rapidly produce microfluidic chips with good dimensional stability. However, the embossing stamp needs to be refabricated each time the microfluidic design is modified. 

The high Young’s modulus of the glass wafer and strong bonding to each other made it the best choice for the inertial focusing and separation of particles, especially at a high flow rate. Therefore, borosilicate glass was used in this study for the fabrication of spiral microfluidic channels. A special Visual Basic for Applications (VBA) code was developed to engrave the trapezoidal-cross-section spiral-channel using ultra-short pulse laser ablation. To evaluate the performance of the developed inertial microfluidics, microparticles of 5 μm, 10 μm, and 15 μm were suspended in the fluid and separated. The results showed that the fabricated microfluidic platform can separate 5 μm particles from the 10 μm and 15 μm particles at an optimized flowrate. Moreover, the developed microfluidic chip can withstand high pressures and flow rates without any deformation, failure, or cracking.

## 2. Materials and Methods

### 2.1. Design and Fabrication of Microfluidic Chips

**Figure 1:**
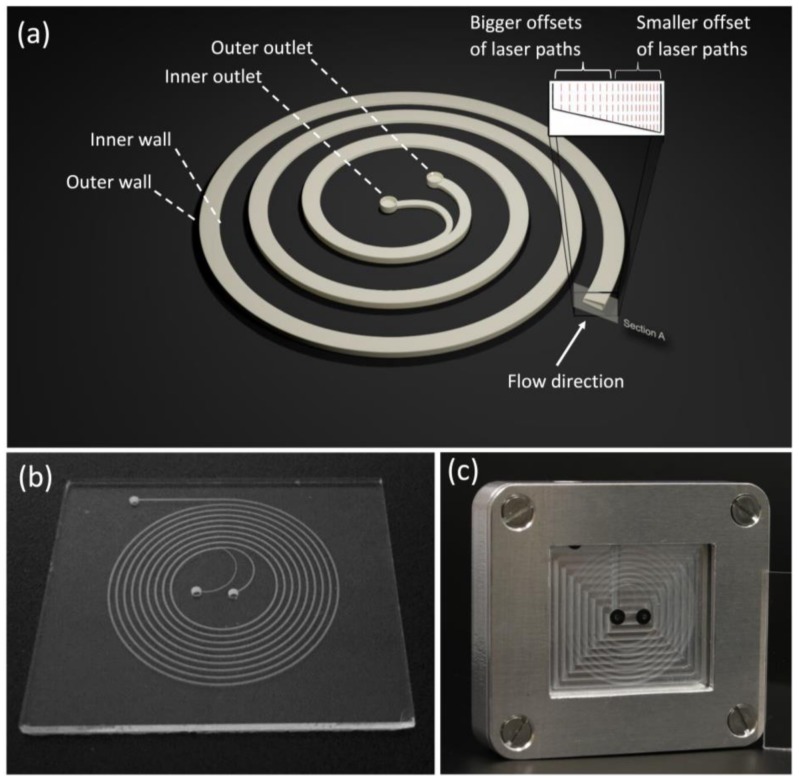
Design of the spiral microfluidic chip and the final fabricated platform (**a**) illustration of the proposed trapezoidal spiral microfluidic channel design with one inlet and two outlets, larger and smaller radial increments as used for the laser ablation providing a sloped channel bottom are illustrated in the insert, (**b**) ready-made and bonded glass platform of the spiral channel, and (**c**) spiral microfluidic chip inside the aluminum holder for easier inlet/outlet tube connections.

In this work, trapezoidal spiral channels for inertial focusing with different aspect ratios were fabricated and tested. Compared to rectangular channels, trapezoidal spiral channels produce Dean vortices with shifted cores towards the outer wall of the spiral channel, as a result, the equilibrium position of small particles is shifted for better separation efficacy. All developed trapezoidal channel designs consisted of 8-loop spirals with one inlet (towards the outer edge of the platform) and two outlets (at the center of the spiral platform) (see Figure 1). The channel was designed with a 600 μm width, and inner/outer heights of 50 and 90 μm, respectively, to form the trapezoid cross-section. Lithography for micro-patterning in glass is a well-developed fabrication method with low cost and simple setup. However, lithography is limited when creating shape features without any 3D features [29]. Therefore, a femtosecond laser was used to directly engrave the targeted designs in glass wafers.

The microfluidic platform features were divided in two groups with different ablation strategies: slicing strategy, and multi-pitch strategy. Inlet/outlet holes and alignment lines for wafer dicing were patterned using a slicing strategy. The trapezoidal channel was created using a multi-line/multi-pitch strategy that was controlled using novel visual basic script. Both groups of features were fabricated in a single 700 μm thick borosilicate glass substrate (BOROFLOAT^®^, Schott AG, Mainz, Germany) thermally bonded to a second unstructured glass wafer as a cover.

A laser workstation (microSTRUCT-C, 3D-Micromac, Chemnitz, Germany) equipped with a Yb:KGW solid state laser (Pharos, Light Conversion, Vilnius, Lithuania) with a maximum average power of 15 W was utilized in this work. Due to the extremely high energy densities during the short pulse durations, multi-photon absorption occurs in materials that usually show no absorption at the laser wavelength, thus practically enabling the structuring of any material by laser ablation. The laser beam with a wavelength of 1030 nm was focused and displaced on the sample surface by means of a galvanometer scanner (intelliScan, SCANLAB, Munich, Germany) equipped with an f-theta lens with a focus length of 100 mm. This setup resulted in a focal spot with a Gaussian intensity distribution and a diameter of around 18 μm. All the components were controlled by the laser platforms microMMI software from 3D-Micromac.

**The slicing strategy for inlet/outlet holes and alignment lines**: this group of micro-features were designed as CAD-files (AutoCAD 2016, Autodesk, San Rafael, CA, USA), then converted into a proprietary laser vector graphics format with the microMMI-software (v3.6.7.2572, Micromag AG, Chemnitz, Germany). The structures to be ablated were sliced into separate layers with a *z*-increment of 50 μm each. For laminar material removal with minimized surface roughness, the laser spot has to be scanned over the geometries in a homogeneous pattern, consisting of sets of parallel lines with a pitch of 8 μm and a margin of 8 μm to the outer contour. Four of these sets were superimposed to form the ablation pattern. Each subsequent set was rotated by 30° against the previous one. Inlet/outlet holes consisted of circular areas with a diameter of 1 mm on 14 slicing layers. The ablation depth of each layer was controlled by adjusting the pulse energy, in this case 76 μJ, which, if using a repetition rate of 100 kHz in combination with a scanning speed of 750 mm/s, removed around 50 μm of material with two repetitions of each layer’s output vectors.

**Multi-line/multi-pitch strategy using visual basic programming for the trapezoidal channels**: Depending on the desired slope of the channel bottom, the use of a slicing strategy is not feasible if a smooth bottom surface is required. The minimal slice thickness depends on the laser parameters, especially on the pulse energy. If pulse energies close to the ablation threshold of the substrate are used, slice thicknesses in the range of a few microns can be achieved. Even though the roughness of a slope, which has been approximated with slice thicknesses in the μm range, may be acceptable, the fabrication time will be quite high due to the high number of required layers. Therefore, the geometry and especially the profile of the laser ablated spiral channels were parametrized as follows: the fabricated spirals were approximations of Archimedean spirals consisting of 16 semi-circle segments, each of which had a constant radius. A visual basic script was used to control the scanning path of the laser. The width of the spiral channel was provided by ablating additional paths equidistant to the original one until the required width was reached. Depending on the profile shape, the script offered two strategies for the ablation of spiral structures: (1) if a flat bottom profile is required, the radial increment between these paths is set to a fixed value of 6 μm; and (2) a sloped profile can be created by linearly increasing the increment from an adjustable minimum value to a maximum of 8 μm over the width of the spiral channel (see Figure 1a). In both strategies, the ablation depth can be controlled by adjusting the pulse energy or the number of repetitions of the ablation strategy.

Starting with a spiral diameter of 5 mm, the diameter of each subsequent semi-circle segment was increased by 0.5 mm against the previous. A channel width of 600 μm width was set as a target of the slope ablation algorithm, which started with a minimal radial increment of 3.3 μm at the deep end of the bottom and a maximum of 8 μm at the shallow end. The ablation, which was performed using a laser repletion rate of 600 kHz, a scanning speed of 1500 mm/s and a pulse energy of 17.6 μJ was repeated six times. At the end of the spiral, two separate outlet channels were fabricated in order to connect to the outlet holes, which were drilled using the previously mentioned slicing method.

After the wafer was fully patterned with all required features, multiple cleaning-steps and the bonding process were conducted. First, the ablated wafer was cleaned using an ultrasonic bath filled with ethanol for 10 min to remove the unwanted ablation dust and glass particles. Sonicated wafers were then dipped in a glass etching solution of 45 mL H_2_O, 100 mL H_3_PO_4_, and 30 mL HF for a duration of 60 s. A detailed study by Erfle et al. [29] showed that the glass etching process produced a cleaner and smother surface at the ablated areas when it was implemented for the optimal duration. Moreover, the dipping process improved the hydrophilicity of the surface from a contact angle of 23° before etching to 5° after etching. A programmed wafer cleaning machine (Fairchild Convac, Neuenstadt, Germany) was then used to clean the etched wafer and the cover wafer. Four cleaning steps were performed by the machine: (1) a 60 s distilled water sprayed at 6 bar pressure and 5500 rpm spinning speed; (2) flooding the wafer surface with a mixture of H_2_SO_4_ + H_2_O for 120 s at 500 rpm; (3) rinse the wafer with water spraying at 500 rpm for 90 s; and (4) dry the wafer at 550 rpm for 15 s. The wafers were then washed under running distilled water and spin dried at high spinning speed. The cleaned wafers were manually aligned and pre-bonded by applying hand pressure. The pre-bonded wafers were loaded in a custom-made press at 4 kN and left in a muffle furnace at 620 °C for 6 h. The bonded 4″ wafers were then diced into four spiral microfluidic platforms (see Figure 1b).

### 2.2. Operational Mechanism

**Figure 2:**
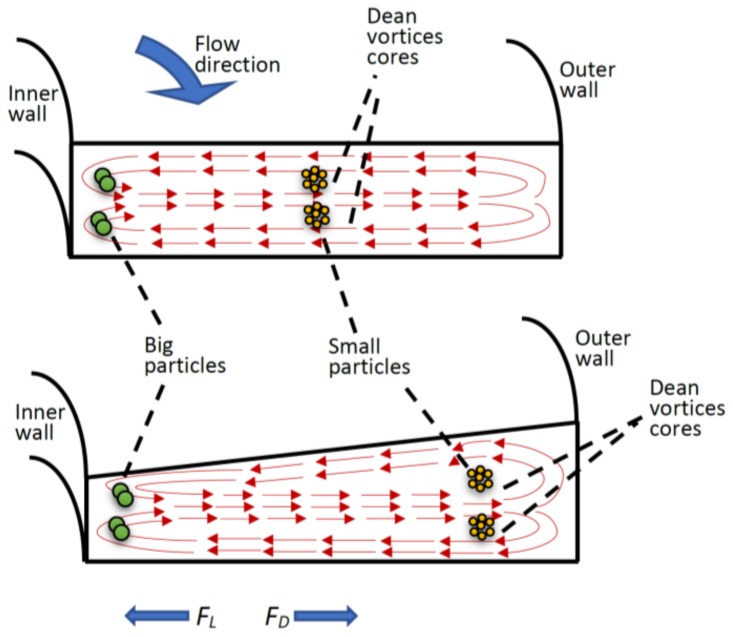
Schematic illustration of the spiral operational concept with demonstration of Dean vortices and equilibrium position of different size particles (**top**) rectangular spiral channel with centered core of Dean vortices, (**bottom**) trapezoidal spiral channel with Dean vortices cores shifted towards the outer wall of the spiral channel, as a result, the equilibrium position of small particles is shifted for better separation efficiency.

In this subsection, the physical phenomena occurring in the spiral microfluidic platforms and the main contributing forces to separate microparticles will be recapitulated. Fluid inertial forces are negligible for many types of microfluidic platforms which are operated at Reynolds numbers below one (*R_e_* = *ρU_f_D_h_/μ*; *ρ* is fluid density, *U_f_* is average fluid velocity, *μ* is dynamic viscosity, and *D_h_* is the channel hydraulic diameter) which falls into the Stokes regime. In contrast, inertial microfluidics operating in the range between the Stokes regime and turbulent regime with *R_e_* typically range from 1 to 100 [7]. Within this operational range, both the inertial and viscosity forces of the fluid influence particle migration in a spiral microfluidic channel. As a consequence of channel curvature shape, two counter-rotating vortices (Dean vortices) develop (see Figure 2). The drag forces resulting from the Dean vortices force the particles to follow the direction of these vortices in addition to the main stream flow. The strength of Dean vortices can be represented by a dimensionless Dean number (*D_e_*) given as [30,31]:

(1) $$D_{e}\;=\;R_{e}\sqrt{\frac{D_{h}}{2R_{C}}\;}$$

where *R_C_* is the radius of spiral channel. Due to the Dean vortices, particles are forced to laterally migrate back and forth between the side walls of the spiral channel. The velocity of this lateral migration of particles is called the Dean velocity (*U_Dean_*) and can be calculated by the following expression [17]:

(2) $$U_{Dean}=1.8\;\times \;10^{-4}D_{e}{}^{1.63}$$

As a particle travels from one side-wall of the channel to the opposite side-wall and then gets back to the initial wall, it is said to have completed a full Dean cycle (*L_DC_*). *L_DC_* can be calculated as follows:

(3) $$L_{DC}=2w+h$$

where *w* and *h* are the channel width and height, respectively. Under the effect of Dean vortices, particles will achieve an equilibrium position within a channel. However, the spiral channel should provide a minimum travel distance for particles to reach the lateral equilibrium position. This minimum travel distance is calculated as follows:

(4) $$l_{C}=\;\frac{U_{F}}{U_{Dean}}L_{DC}$$

where *U_F_* is the average fluid velocity in axial direction. Finally, Stokes’ law can be used to calculate the Dean drag force (*F_D_*) as follows:

(5) $$F_{D}=3\pi \mu U_{Dean}a_{c}$$

where *a_c_* is particle diameter. In spiral channels, the curved geometry produces another force that affects the flowing particles, called the inertial lift force (*F_L_*). *F_L_* is the balance of shear gradient lift force (*F_LS_*) and wall induced lift force (*F_LW_*), and acts on particles in the opposite direction of the Dean drag force [9,31] (see Figure 2). Inertial lift force can be calculated as [10]:

(6) $$F_{L}=\;\frac{2\rho U_{F}{}^{2}a_{c}{}^{4}}{D_{h}{}^{2}}$$

In this work, trapezoidal spiral channels were utilized for complete separation of microparticles based on size. Small sized particles were transposed by the Dean drag force towards the outer wall of the channel (focused around the Dean vortices core, see Figure 2). Bigger particles, however, found lateral equilibrium positions closer to the inner wall of the channel as a consequence of the Dean drag force coupled with inertial lift force. Therefore, particles of different sizes were focused in distinct streams and could be collected from different outlet channels. The ratio of inertial lift force to the Dean drag force, *R_f_ = a_c_*^3^*R_c_*/*D_h_*^3^ determines the cross-sectional equilibrium position of particles. As *R_f_* approached 0 (when particle size is much smaller than channel hydraulic diameter), the Dean drag force is dominant and will drive particles towards the outer wall of the spiral channel. In contrast, as *R_f_* approaches ∞ (when particle size is more comparable to channel hydraulic diameter), inertial force is dominant, and particles will be focused closer to the inner wall of the spiral channel. Therefore, particles of different sizes will be passively separated and focused in different positions of the channel cross-section.

## 3. Results and Discussion

In this subsection, the fabrication and experimental results are presented and discussed. In the fabrication results section, the parameters of laser-ablation that were implemented to produce the suitable microfluidic channels are discussed. In the second section, the separation results using the fabricated microfluidic chip are presented.

### 3.1. Fabrication Results

**Figure 3:**
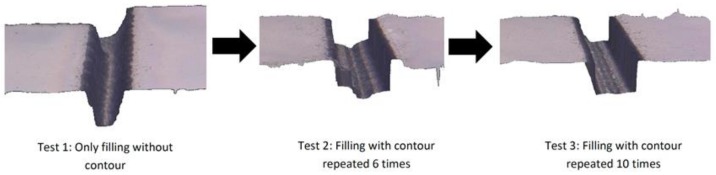
Example illustrating the effect of the laser contouring process (**left**) microchannel without contouring, (**middle**) microchannel with contouring depth less than channel engraving depth (**right)** contouring with adequate number of repetitions (depth).

In this work, multiple iterations were performed to fabricate the required microfluidic design with the best and most accurate results possible. The goal was to realize the required channel slope, perpendicular walls, lowest surface roughness, and correct dimensions. To obtain the required slope (90/50 μm depth), radial increments were varied from 6 μm from the inner wall side to 0.2 μm towards the outer wall side across the channel width (see Figure 1a). For realizing perpendicular walls and overcoming the limitation provided of the conical-shape of the laser beam, a contouring process was directly performed after engraving the microfluidic channel. Contouring is the process of running a straight laser path along the boundary of microfeatures with the offset toward the center of the feature equal to the radius of laser beam (around 8 μm in our case). Figure 3 shows how the contouring process with the right depth (by repetition) can help to produce microchannels with perpendicular walls.

**Figure 4:**
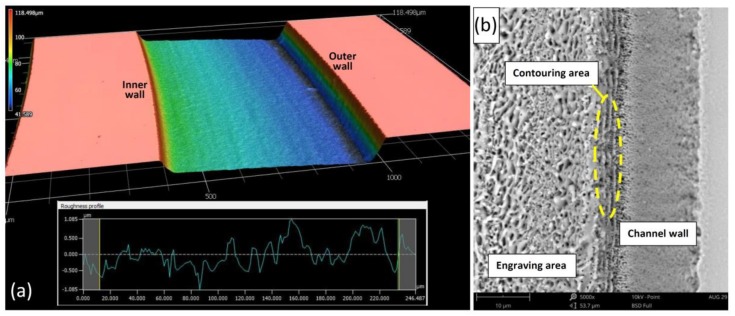
The fabricated spiral channel (**a**) 3D CLSM image of the fabricated channel with the roughness profile of the engraved area (**b**) 5000x SEM image of the fabricated channel showing the texture of the engraved, contouring, and wall areas of the channel.

For acceptable channel roughness, a set of homogenous laser ablations was performed for the whole microchannel. Each subsequent set was rotated 30 degrees in reference to the previous set. Figure 4a,b show the roughness profile and scanning electron microscope (SEM, Phenom-World, Eindhove, The Netherlands) image of the fabricated microchannel. With the multi-ablation set strategy, a roughness of 0.34 μm (*Ra*) was obtained directly after the laser ablation process. After performing chemical etching and thermal bonding, roughness was reduced to around 0.27 μm. Chemical etching also helped to partially remove molten glass filaments and sticking dust.

### 3.2. Experimental Results

To evaluate the proposed spiral microfluidic channel, the focusing and separation of 5, 10, and 15 μm polystyrene fluorescent (emission peak at 502/518 nm) particles (microParticles GmbH, Berlin, Germany) were implemented. For each experiment, 20 μL from each initial particle size solution was diluted in 10 mL of deionized water and mixed with lab vortex for 2 min. To experimentally determine the equilibrium position of each particle size, each diluted sample was loaded in a 10 mL syringe and mounted on a syringe pump (Kd Scientific KDS200, Holliston, MA, USA) and tested with a range of flow rates. An inverted microscope (Olympus CKX53, Tokyo, Japan) equipped with a beam splitter (505 mm) set and broadband blue excitation (460–495 nm) for the excitation of fluorescence and high-speed camera was utilized to observe particle focusing.

**Figure 5:**
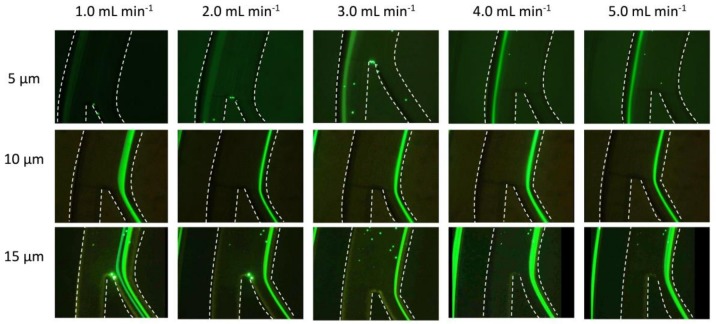
Microparticles focusing paths vs. flow rate of fluid in the proposed trapezoidal spiral channel (dashed lines used to highlight microchannel walls).

**Figure 6:**
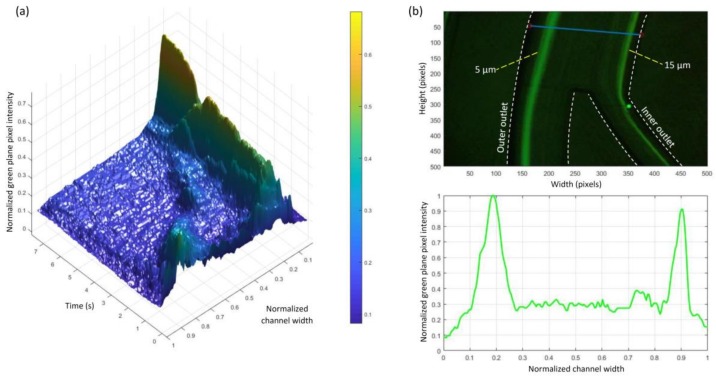
(**a**) 3D green-plane intensity profile of experimentally focusing 5 μm particles when increasing flow rate from 0 to 5 mL·min^−1^ over a period of around 8 s; (**b**) 5 μm and 15 μm particles separation using the proposed trapezoidal spiral channel (**top**) image captured with the inverted fluorescence microscope at 5 mL·min^−1^ (**bottom**) green intensity profile shows the focusing peaks (each block on the *x*-axis is equal to 60 μm of the real channel width).

Prior to the experiments, we performed mathematical calculations to evaluate the proposed dimension of the spiral channel. Table 1 shows the calculated Dean number, *F_D_*, and *F_L_* for the implemented particle sizes and flow rates (according to Equations (1), (5), and (6)). It can be seen that for the 5 μm particle, *F_D_* was always higher than *F_L_* for the whole range of implemented flow rates. On the other hand, *F_L_* was always higher than *F_D_* for the 10 and 15 μm particles. This confirmed our expectation that the *F_D_* force would be dominant for the 5 μm particles and force them to focus at the core of the Dean vortices near the outer wall of the channel. In contrast, *F_L_* was the dominant for both 10 and 15 μm particles, therefore, they focused at flow paths near the inner wall of the spiral channels. These expectations were fulfilled in our experiments as shown in Figure 5 and Figure 6.

The fabricated microfluidic chips were initially mounted to the inverted microscope and primed with deionized (DI) water for 2 min to force out any air bubbles or particles. After the priming step, the prepared solution of each particle size was injected separately and the results recorded. Figure 5 shows the experimental results obtained with particles of different sizes at flow rates increased in steps of 1 mL·min^−1^ from 1 to 5 mL·min^−1^. It was obvious that the 5 μm particles focused near the outer wall of the spiral channel and were recovered from the outer outlet. Ten and 15 μm particles were focused near the inner wall and recovered from the inner outlet of the spiral channel. To test how different flow rates could affect the focusing point of microparticles, the flow rate was varied while recording the migration of 5 μm particles at the segment just before the outlet junction. First, the channel was primed with the prepared microparticles sample, then flow rate was increased from 0 mL·min^−1^ to 5 mL·min^−1^ over a period of around 8 s. Figure 6a shows a 3D profile of the green intensity across the channel width for this experiment. It can be seen that the 5 μm particles focused on two paths at a low flow rate. The two paths unified on the focusing path near the outer wall when the flow rate increased to the more suitable flow rate of >1.0 mL·min^−1^. This test demonstrates how the flow rate could significantly affect the position of the particles focusing path. Finally, a mixture of 5 μm and 15 μm particles was injected at a 5 mL·min^−1^ flow rate, and the result is presented in Figure 6b. It can be seen that the particles focused in two distinct paths with a gap between them of around 420 μm (70% of channel width). This relatively big gap could produce a higher purity of separated particles when recovered from the two outlets of the spiral channel.

## 4. Conclusions

In this work, a femtosecond laser was utilized as a fast prototyping method to fabricate spiral microfluidic channels with a trapezoidal cross-section. In contrast to previously investigated spiral channels for particle separation, a novel spiral channel platform was fabricated in a 0.7 mm glass wafer for higher optical transparency, improved thermal resistivity, and better dimensional stability than those provided by PDMS platforms. Moreover, the femtosecond laser ablation could overcome the limitation of chemical etching when it comes to fabricating microfluidic structures with three dimensional features. To fabricate the trapezoidal cross section, a special VBA code was developed with wide flexibility to fabricate any slope required. The fabrication results showed good dimensional accuracy and low surface roughness. The surface roughness was further improved by subsequent chemical etching and thermal bonding. The focusing and separation of 5 μm, 10 μm, and 15 μm particles were performed to evaluate the proposed spiral channel. Moreover, from a mixture of 5 μm and 15 μm particles, both types of particles could be separated and recovered from the two outlets of the spiral channel. The experiments showed relatively big separation gaps of around 420 μm between the particle paths that also improved the purity of the separated particles. By fabricating the spiral channel in a glass substrate, high flow rates were achieved with the ability to process 3 mL of sample per minute. In conclusion, the new laser fabrication method could be implemented as an accurate and fast prototyping process for the fabrication of 3D spiral channels. These spiral channels can potentially be implemented in commercial biological processes where different sized cells need to be separated, i.e., red blood cells and circulating tumor cells. Moreover, the improved chemical inertness can widen the range of applicable processes for the glass microfluidic chips such as the detection of specific chemical components in the environment, food, and medical samples.

## Figures and Tables

**Table 1:** Calculated Dean forces and inertial lift forces for different particle sizes and flow rates.

Particle Size *a_c_* (μm)	Flow Rate (mL·min^−1^)	Dean Number *D_e_*	*F_D_* (*N*)	*F_L_* (*N*)
5	1	5.46	1.3 × 10^−10^	1.2 × 10^−11^
	3	16.6	8.3 × 10^−10^	1.1 × 10^−10^
	5	27.7	1.9 × 10^−9^	3.1 × 10^−10^
10	1	5.46	2.7 × 10^−10^	1.9 × 10^−10^
	3	16.6	1.6 × 10^−9^	1.8 × 10^−9^
	5	27.7	3.8 × 10^−9^	4.9 × 10^−9^
15	1	5.46	4.0 × 10^−10^	9.8 × 10^−10^
	3	16.6	2.4 × 10^−9^	9.1 × 10^−9^
	5	27.7	5.7 × 10^−9^	2.5 × 10^−8^
